# Whole-Genome Sequences of Two Strains of Porcine Circovirus 2 Isolated from Calves in Germany

**DOI:** 10.1128/genomeA.01150-13

**Published:** 2014-01-09

**Authors:** Mohammad Yahya Halami, Hermann Müller, Jens Böttcher, Thomas W. Vahlenkamp

**Affiliations:** Institute of Virology, Center for Infectious Diseases, Faculty of Veterinary Medicine, University of Leipzig, Leipzig, Germanya; Bavarian Animal Health Service, Poing, Germanyb

## Abstract

Two new strains of porcine circovirus type 2 virus (PCV2), strains Ha09 and Ha10, were detected in calves in Germany, and the complete genome of each virus has been sequenced and analyzed. Phylogenetic analysis suggests that these strains belong to the PCV2b genotype cluster, a highly prevalent genotype found worldwide.

## GENOME ANNOUNCEMENT

Porcine circovirus type 2 (PCV2), which belongs to the family *Circoviridae*, is a nonenveloped, icosahedral, single-stranded circular DNA virus with a genome of about 1,768 nucleotides (nt). PCV1 and PCV2 are the smallest viruses that replicate autonomously in mammalian cells (1). The PCV2 genome contains two major open reading frames (ORF1 and ORF2) encoding for the replication (Rep) and capsid (Cap) proteins (2), respectively, as well as a third gene (ORF3) encoding a virus-induced apoptotic protein (3). PCV2, identified as the etiologic agent of porcine circovirus-associated diseases (PCVAD), causing severe economic losses (4), was also detected in rodents (5), cattle (6), and in calves affected with bovine neonatal pancytopenia (BNP) (7). There are three different recognized PCV2 genotypes: PCV2a, PCV2b, and PCV2c (8, 9). In this study, two PCV2 strains were detected and identified in calves affected with BNP in Germany. During the screening for the prevalence of PCV2 in calves, we investigated 181 EDTA-blood and tissue samples. Two out of 181 samples were found to be positive for PCV2, both of which were from calves with signs that are typical for BNP. However, the first strain, designated Ha09, originated from the blood of a calf in Bavaria, whereas the second strain, Ha10, was isolated from the lung and brain of a calf in Saxony. The DNA was extracted from the samples using the High Pure PCR template preparation kit (Roche, Mannheim, Germany), and a nested broad-spectrum PCR protocol was applied, as recently described (10). The complete genome of each detected circovirus was amplified by PCR using a pair of inverse primers that were previously described (7). Each whole genome was amplified and subsequently cloned and sequenced.

The genome of the strain Ha09, which originated from Bavaria in the south of Germany, is composed of 1,768 nt; however, strain Ha10, which was collected in Saxony in the east of Germany, contains 1,767 nt. Both strains consist of 3 ORFs (ORF1, ORF2, and ORF3). The level of identity between the complete sequences of the Ha09 and Ha10 strains is 98.8%. These strains are more closely related to the PCV2b types than to the other genotypes, PCV2a and PCV2c. The identity scores to the PCV2b type based on the complete sequence of the Ha09 and Ha10 are 98.9% and 99.6%, respectively. A phylogenetic tree based on capsid nucleotide sequences revealed that the isolate from Saxony (Ha10) is more closely related to PCV2b than the isolate from Bavaria (Ha09). Compared to the published PCV2b sequences, a detailed analysis of the capsid amino acid sequences revealed four identical amino acid changes in the isolate from Bavaria and 2 others in the isolate from Saxony. According to the type of these amino acids, it may be expected that these changes may not have any significant influence on the biological or antigenic characteristics of these isolates. This finding was further supported by phylogenetic analysis of the replication protein sequences. The obtained data will be helpful for analyses of the evolutionary characteristics and molecular pathogenesis of PCV2 and its prevalences in nonporcine hosts.

### Nucleotide sequence accession numbers.

The genome sequences of the Ha09 and H10 strains have been submitted to GenBank under accession no. HQ231329 and HQ231328, respectively. The GenBank numbers of the reference strains are AF055394 for PCV2b, AF055392 for PCV2a, and EU148503 for PCV2c.
